# Postoperative Drainage Efficacy of 8Fr Pigtail Catheter After Bullectomy: A Non-Inferiority Study

**DOI:** 10.7759/cureus.73596

**Published:** 2024-11-13

**Authors:** Masayoshi Hirohara, Shingo Ochiai, Hashimoto Tomoya, Tadao Kubota

**Affiliations:** 1 General Surgery, Tokyo Bay Urayasu Ichikawa Medical Center, Urayasu, JPN

**Keywords:** bullectomy, chest tube, pigtail catheter, primary spontaneous pneumothorax, video-assisted thoracoscopic surgery (vats)

## Abstract

Background

There are no established guidelines regarding the optimal size of chest tubes following a bullectomy. While large chest tubes are commonly used after bullectomy, several studies have shown that pigtail catheters can be effectively employed for postoperative drainage in lung cancer surgery. This study aimed to compare the time to tube removal between an 8Fr pigtail catheter and a 24Fr chest tube after bullectomy to assess the non-inferiority of the 8Fr pigtail catheter.

Methods

Data from 32 patients aged 14-30 years who underwent bullectomy between April 2020 and April 2023 were analyzed. Participants were assigned to receive either an 8Fr pigtail catheter (n = 10) or a 24Fr chest tube (n = 22). The primary outcome measured was the number of days until tube removal.

Results

The mean time to tube removal was 1.2 days in the 8Fr group and 1.5 days in the 24Fr group, meeting the non-inferiority margin of one day (mean difference: -0.3 days, 95% CI: -0.78, 0.18). No major complications were observed in either group.

Conclusions

An 8Fr pigtail catheter appears to be a non-inferior alternative to the 24Fr chest tube for postoperative drainage following bullectomy.

## Introduction

Primary spontaneous pneumothorax (PSP) affects up to 28 individuals per 100,000 population annually [1]. Once PSP occurs, the recurrence rate is estimated to range between 25% and 43%, with bullectomy recommended as a preventive measure [2-5]. Post-bullectomy, a chest tube is commonly placed; however, research on its management is limited. In 2017, Pompili et al. recommended that a chest tube after bullectomy should typically be 24 or 28 Fr, maintained under negative pressure at -20 cmH2O for 48 hours [6].

Recently, with the low complication rate of bullectomy, several studies have explored less invasive chest tube management strategies. A 2021 study by Kawaguchi et al. demonstrated that chest tubes could be feasibly removed in the operating room if air leaks ceased and full lung re-expansion was confirmed [7]. While much of the research has focused on the timing of chest tube removal, few studies have specifically examined tube size.

In thoracoscopic lung resection for lung cancer, some studies have investigated postoperative chest tube size and shown that pigtail catheters (8-12Fr) are a feasible alternative to larger conventional chest tubes [8-10]. Pigtail catheters have also been effective in a variety of other applications, such as drainage for pneumothorax and hemothorax, further supporting their viability as an alternative to traditional larger chest tubes [11-16]. Based on these findings, we assessed our approach to chest tube use after a bullectomy. While a 24Fr chest tube had traditionally been placed, we hypothesized that the 8Fr pigtail catheter could be a viable alternative.

A potential concern with the 8Fr pigtail catheter, compared to the 24Fr chest tube, is its smaller diameter, which may reduce drainage efficiency. The smaller size could delay lung re-expansion and the resolution of postoperative air leaks, potentially extending the time before tube removal. Therefore, in this study, we aim to compare the 8Fr pigtail catheter with the 24Fr chest tube to determine whether the use of the 8Fr pigtail catheter results in a longer duration of tube removal after bullectomy. We have established a non-inferiority margin of one day, meaning that if the time to remove the 8Fr pigtail catheter is not delayed by more than one day, we can conclude that its postoperative drainage performance is not inferior to that of the 24Fr chest tube. Comparisons of postoperative pain were not included, as the use of analgesics was not standardized.

## Materials and methods

Data were extracted from the Fujitsu electronic medical records system to create a study database covering the period from April 2020 to April 2023. This database included patient demographics, baseline characteristics, and outcome measures. Participants were aged between 14 and 30 years and underwent surgery to prevent recurrent PSP. Preoperative assessments included blood tests, chest X-rays, and CT scans. During surgery, three small incisions were made in the chest wall, and a thoracoscope was inserted through one of the incisions to visualize the bullae. The bullae were resected using an Endo GIA stapler (Medtronic, Dublin, Ireland), and a chest tube was inserted after confirming that there was no air leak from the lung.

From April 2020 to March 2022, a 24Fr chest tube was inserted, and from April 2022 to April 2023, an 8Fr pigtail catheter was inserted, both manufactured by Cardinal Health. Postoperatively, patients were monitored in the surgical ward. Starting from postoperative day (POD) 1, daily assessments were conducted, and the chest tube was removed once the following criteria were met: absence of air leaks and adequate lung re-expansion on chest X-ray. A follow-up chest X-ray was performed the day after chest tube removal, and if lung expansion was confirmed, the patient was scheduled for discharge the following day.

The primary outcome of the study was the number of days from surgery to chest tube removal. A non-inferiority margin of less than one day in the mean number of days to chest tube removal was defined. Secondary outcomes included postoperative length of stay and the occurrence of postoperative complications (Clavien-Dindo grade ≥2).

In this non-inferiority study, the sample size was calculated using EZR software (Saitama Medical Center, Jichi Medical University, Saitama, Japan). A significance level of 0.05 and a statistical power of 80% were assumed. The sample size calculation indicated that a total of eight participants would be required, with four participants in each group. Non-inferiority was assessed using the one-sided 95% CI approach for the primary outcome. Continuous variables were analyzed after assessing normality using the Shapiro-Wilk test. Normally distributed data were analyzed using the t-test, while non-normally distributed data were assessed with the Wilcoxon test. Fisher’s exact test was used for nominal variables. A p-value of less than 0.05 was considered statistically significant. All statistical analyses were performed using EZR software. The study was approved by the Institutional Review Board of the Tokyo Bay Urayasu Ichikawa Medical Center (approval number 857). As a retrospective study, informed consent was waived, and patient confidentiality was maintained through data anonymization.

## Results

A total of 32 patients were included in the study, with 10 assigned to the 8Fr pigtail catheter group and 22 to the 24Fr chest tube group. There were no significant differences between the two groups in terms of sex, age, height, BMI, operative duration, smoking history, and recurrent pneumothorax (Table 1).

**Table 1:** Patient demographics and characteristics This table summarizes the demographic data of the participants, including sex, age, height, BMI, operative duration, smoking history, and recurrent PSP. Data are expressed as frequency (percentage) for categorical variables and mean ± SD for continuous variables. PSP, primary spontaneous pneumothorax

Parameter	8Fr (n = 10)	24Fr (n = 22)	t-statistic	W-statistic	p-value
Male sex	9 (90)	21 (95)			0.53
Age (years)	18.9 ± 4.1	21.1 ± 3.8	1.41		0.17
Height (cm)	173.3 ± 3.6	170.2 ± 9.8		128	0.48
BMI (kg/m^2^)	18.5 ± 2.2	18.4 ± 2.3	0.13		0.89
Operative duration (min)	55.3 ± 12.6	49.3 ± 18.4	1.08		0.29
Smoking history	0 (0)	3 (13.6)			0.53
Recurrent PSP	8 (80)	13 (59.1)			0.43

**Figure 1:**
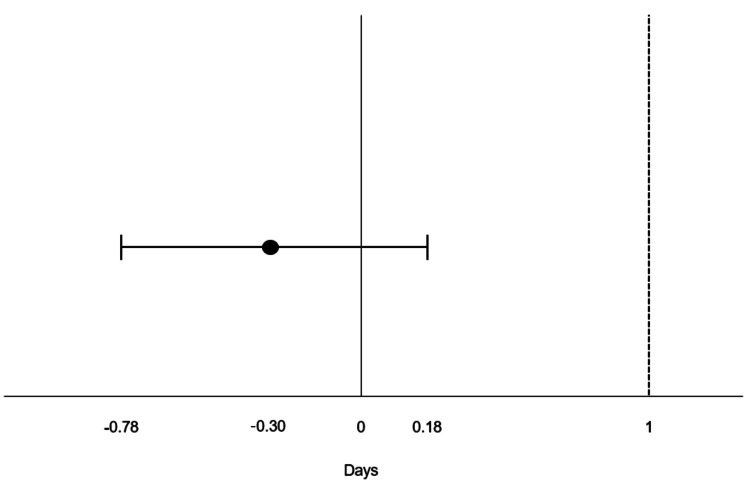
95% CI for the difference in mean days to chest tube removal The dashed line represents the prespecified non-inferiority margin of one day, and the circle indicates the mean difference in days to tube removal. The horizontal line represents the 95% CI.

The primary outcome, the number of PODs until chest tube removal, was 1.2 days (95% CI: 0.90, 1.50) for the 8Fr group and 1.5 days (95% CI: 1.20, 1.80) for the 24Fr group. The mean difference in days between the two groups (8Fr minus 24Fr) was -0.3 days (95% CI: -0.78, 0.18), which met the prespecified non-inferiority margin of one day (Figure 1). A multiple linear regression analysis, using tube size, smoking history, and operative time as explanatory variables for the days to tube removal, revealed no significant differences (Table 2).

**Table 2:** Results of multiple regression analysis regarding the days to tube removal This table shows the estimated coefficients, SEs, and p-values for the predictors associated with days to tube removal in the study population. Explanatory variables include tube size, smoking history, and operative duration.

Parameter	Estimated coefficient	SE	p-value
Tube size (24 Fr)	0.39	0.24	0.11
Smoking history (Yes)	-0.19	0.37	0.59
Operative duration (min)	0.11	0.01	0.11

Regarding secondary outcomes, the mean postoperative length of stay was 2.8 ± 0.8 days for the 8Fr group and 3.5 ± 1.1 days for the 24Fr group (p = 0.063). No postoperative complications were observed in either group (Table 3).

**Table 3:** Comparison of postoperative length of stay and complications This table shows the mean length of hospital stay and the incidence of postoperative complications (Clavien-Dindo ≥2) for patients in the 8Fr and 24Fr groups. Data are expressed as mean ± SD for length of stay and frequency (percentage) for complications.

Parameter	8Fr	24Fr	W-statistic	p-value
Postoperative length of stay	2.8 ± 0.8	3.6 ± 1.1	66	0.063
Postoperative complications	0 (0)	0 (0)		

## Discussion

The aim of this retrospective study was to evaluate the safety of using an 8Fr pigtail catheter compared to a 24Fr chest tube for postoperative drainage following bullectomy. Our findings indicated that the 8Fr pigtail catheter was non-inferior to the 24Fr chest tube in terms of time to tube removal, with a mean difference of -0.3 days (95% CI: -0.78, 0.18), which falls within the prespecified non-inferiority margin of one day. These results suggest that the 8Fr pigtail catheter can serve as a viable alternative to the 24Fr chest tube with comparable clinical outcomes.

Several limitations should be acknowledged. First, the retrospective nature of the study, relying on preexisting records, may have introduced inaccuracies. Second, the absence of randomization between groups may have led to bias in the comparison, affecting the interpretation of causal relationships. Finally, the fact that group allocation was primarily determined by the surgeon is a notable limitation, as surgeon preferences or clinical decisions may have influenced group assignment.

Despite these limitations, this study holds significant value, particularly as few studies have explored the impact of chest tube size following bullectomy. Traditionally, larger diameter chest tubes, such as 24Fr and 28Fr, have been recommended; however, the 8Fr pigtail catheter has emerged as a feasible option. The 8Fr pigtail catheter offers two advantages: it causes less pain during insertion and eliminates the need for suturing at the insertion site. Although postoperative pain was not assessed in this study due to the lack of standardized analgesia use, previous studies have reported that small-bore catheters reduce pain during insertion compared to larger-bore catheters [17,18]. This reduction in pain is thought to result from less pressure on the intercostal nerves, facilitating earlier mobilization and discharge. Additionally, the smaller insertion site associated with the 8Fr pigtail catheter eliminates the need for suturing, which is required when using a 24Fr chest tube.

One concern with small-bore catheters is that their drainage efficiency may be inferior to that of large-bore catheters, potentially leading to a longer duration of tube removal. However, previous studies using 12Fr pigtail catheters after lung resection have not shown a significant difference in drainage duration compared to large-bore (20-32Fr) catheters [9]. Furthermore, studies using small-bore (≦14Fr) pigtail catheters for pneumothorax treatment have demonstrated a significant reduction in drainage duration compared to larger catheters (≧16Fr) [11]. In this study, no significant difference was observed in the mean number of days to tube removal between the 8Fr pigtail catheter and the 24Fr chest tube, aligning with previous research. Regarding postoperative complications, persistent air leaks, and postoperative bleeding have rarely been reported after bullectomy, and the findings of this study were consistent with these reports [19,20].

Future research should focus on larger prospective studies with increased sample sizes to determine whether the 8Fr pigtail catheter offers advantages over traditional larger-diameter chest tubes in terms of postoperative pain and length of hospital stay.

## Conclusions

This study demonstrated that the 8Fr pigtail catheter is a non-inferior alternative to the traditional 24Fr chest tube for postoperative drainage following bullectomy in patients with PSP. The primary outcome, time to chest tube removal, was comparable between the two groups, with a mean difference falling within the pre-specified non-inferiority margin of one day. These findings suggest that the 8Fr pigtail catheter offers a safe and effective alternative to larger chest tubes.

Additionally, the 8Fr pigtail catheter presents potential advantages, such as reduced pain during insertion and the elimination of the need for suturing the insertion site after removal. While the results of this study are promising, future research involving larger, randomized trials is required to further evaluate the benefits of the 8Fr pigtail catheter, particularly with regard to postoperative pain and length of hospital stay.
